# Trace Element Selenium Effectively Alleviates Intestinal Diseases

**DOI:** 10.3390/ijms222111708

**Published:** 2021-10-28

**Authors:** Ruihua Ye, Jiaqiang Huang, Zixu Wang, Yaoxing Chen, Yulan Dong

**Affiliations:** 1 College of Veterinary Medicine, China Agricultural University, Beijing 100193, China; 15036411345@163.com (R.Y.); zxwang2007@163.com (Z.W.); yxchen@cau.edu.cn (Y.C.); 2 Key Laboratory of Precision Nutrition and Food Quality, Ministry of Education, China Agricultural University, Beijing 100193, China; bornhuang@foxmail.com; 3 Beijing Advanced Innovation Center for Food Nutrition and Human Health, Department of Nutrition and Health, China Agricultural University, Beijing 100193, China

**Keywords:** selenium, selenoproteins, intestinal diseases, IBD, SeNPs

## Abstract

Selenium (Se) is an essential trace element in the body. It is mainly used in the body in the form of selenoproteins and has a variety of biological functions. Intestinal diseases caused by chronic inflammation are among the most important threats to human health, and there is no complete cure at present. Due to its excellent antioxidant function, Se has been proven to be effective in alleviating intestinal diseases such as inflammatory bowel diseases (IBDs). Therefore, this paper introduces the role of Se and selenoproteins in the intestinal tract and the mechanism of their involvement in the mediation of intestinal diseases. In addition, it introduces the advantages and disadvantages of nano-Se as a new Se preparation and traditional Se supplement in the prevention and treatment of intestinal diseases, so as to provide a reference for the further exploration of the interaction between selenium and intestinal health.

## 1. Introduction

Selenium (Se) is an essential trace element with biological functions that are important for human health. It was first discovered in 1817 [1]. In the 1950s, it was found that the accumulation of Se leads to myocardial dystrophy or acute liver necrosis. Studies have shown that Se-deficient areas are more prone to endemic cardiomyopathy, whereas dietary supplementation of Se can protect liver necrosis. Since then, there has been substantial interest in the biological role of Se, and researchers have begun to explore the biochemical role of Se [2,3,4,5,6]. Se is necessary for the function of the immune system, and Se deficiency is associated with a loss of immune capacity [7]. Se is indispensable for the brain. Se deficiency causes irreversible brain damage during Se depletion [8]. Se also plays an important role in the maintenance of intestinal health [9]. Dietary Se supplementation can significantly alleviate the degree of IBD, Se can improve the antioxidant capacity of organisms through different pathways, and Se can relieve inflammation by enhancing the antioxidant function of the intestinal tract [10]. The main source of Se in the body is from the diet. The main dietary sources of Se are bread and cereals, meat, fish, eggs, and milk/dairy products [11]. In addition, with the increasing attention being paid to Se, researchers have explored obtaining Se from dietary supplements in recent years, and Se is often included in multivitamin/mineral supplements [12,13].

In recent years, IBD has been one of the factors threatening human health [14]. Se has a good antioxidant function, which can reduce the production of reactive oxygen species (ROS) to a large extent, reduce the damage to the intestinal mucosa, and improve the ability of intestinal oxidation [15,16]. In addition, it has been shown that Se supplementation can also reduce the effect of intestinal damage through autophagy and apoptosis pathways [17,18]. This paper reviews the role of Se in the body, particularly the role of Se in regulating intestinal diseases and the mechanism of action, and it introduces a new type of Se preparation: nano-Se particles, which can significantly improve its bioavailability and antioxidant ability. Therefore, this review provides a reference for further study of the relationship between Se and the intestinal tract to some extent.

## 2. Se and Selenoproteins Have a Close Connection with the Organism

### 2.1. Basic Information about Se

**Figure 1:**
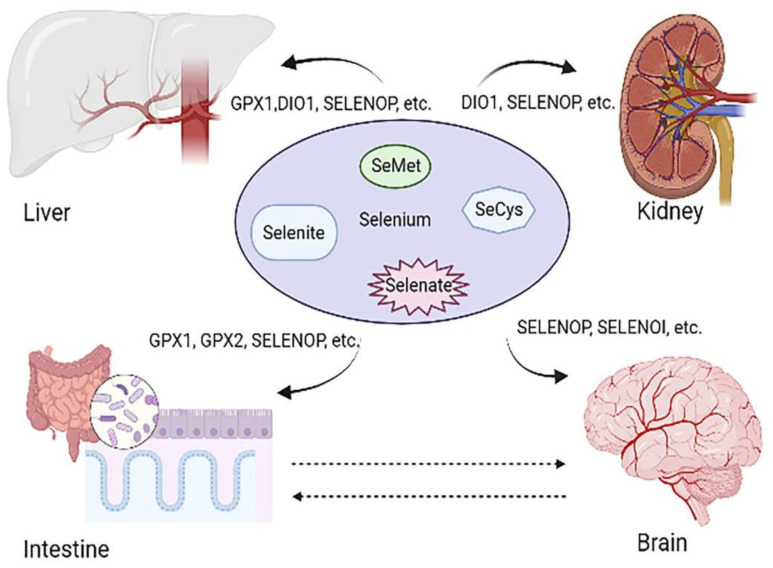
Different organs are regulated by different selenoproteins. Se plays an important role in maintaining body health and can regulate body health through oxidative stress and immune regulation. Se usually exists in the form of selenite, Secys, SeMet and selenate. Se is absorbed in the body mainly in the form of selenoproteins. GPX1 and GPX2 can regulate body health by mediating the production of reactive oxygen species. SELENOP can be expressed as a plasma transporter in various organs, and it is downregulated in cancer and IBD. DIO1 can affect the metabolism and activity of thyroid hormones. DIO1 deficiency contributes to kidney cancer, and DIO1 overexpression inhibits the proliferation and migration of renal cancer cells. SELENOI regulates body health through the nervous system. Loss of SELENOI promotes neurodegenerative diseases such as Alzheimer’s disease and hereditary spastic paraplegia.

The trace element selenium (Se) was discovered in 1817 by the Swedish chemist Berzelius, who named it after the moon goddess, Selene, in Greek mythology [1,6,19]. Se is an essential trace element with biological functions of utmost importance for human health. Se level can affect major organ health, as can be observed in Figure 1. Unlike other metals, it is incorporated into proteins by means of a co-translational mechanism as part of the amino-acid selenocysteine (SeCys), the 21st amino acid used for proteins synthesis in humans [2]. Twenty-five selenoproteins have been identified so far in humans, whereas only a few of them have been functionally characterized [19,20]. Se consumed in foods and supplements exists in a number of organic and inorganic forms, including selenomethionine (plant and animal sources and supplements), selenocysteine (mainly animal sources), selenate, and selenite (mainly supplements) [21]. Detailed information can be seen in Table 1. Se is present in nature and organisms as organic and/or inorganic forms. The main organic forms are selenomethionine (SeMet) and selenocysteine (Secys) [22]. The inorganic forms are selenite (SeO_3_^2−^), selenide (Se^2^^−^), selenate (SeO_4_^2−^), and elemental Se (Se) [23]. Inorganic Se is prone to combining with vitamins, and it has poor stability and low bioavailability. In addition, inorganic Se’s toxicity is relatively high, with difficulty in controlling the dose; it can cause severe effects if the dose in the body is partially excessive. Compared with inorganic Se, organic Se has a high stability and is easy to be absorbed and be used by organisms. It is a safe and effective way to supplement Se. After being absorbed by the organism it can be quickly used by the human body to effectively improve the human body’s blood Se level [19].

Se is present in a wide variety of foods. The Se content in foods depends on the Se concentration in soil where plants are grown or animals are raised, which leads to tremendous variation in Se [24]. Animal-based foods (e.g., pork, beef, mutton, chicken, fish, milk, and egg) are the best sources of Se (mostly selenocysteine) for humans [25]. In addition, liver, shellfish, and fish are moderately good sources. The main route for Se intake is via the diet, whereas the contribution from water and air is negligible [26,27,28,29]. An estimation of Se levels in different types of food was recently reviewed [30,31,32,33]. The distribution of Se was diverse in different areas. The northeast and southwest regions of China are low-Se areas, whereas high-Se areas are mainly distributed in the eastern and southern regions of China. Se deficiency can lead to Keshan disease and Kashin–Beck disease (KD and KBD); thus, daily Se is necessary. Therefore, the average recommended daily intake of Se for adults is 53 μg per day for women and 60 μg per day for men [33,34,35,36,37,38,39].

### 2.2. Selenoproteins: The Main Form of Se Utilization

The physiological functions of Se are thought to result from its existence in a number of selenoproteins in which Se is present as the amino acid selenocysteine (Sec) [1,40,41]. Se was first shown to be an essential component of glutathione peroxidase and has subsequently been found (or predicted to be found) in 25 mammalian selenoproteins [42,43]. Se in the human body mainly plays a protective role against oxidative damage, regulating immune function, and inhibiting inflammatory response in the form of selenoproteins [44]. Sec is incorporated into the amino-acid sequence of selenoproteins during translation, being coded for by a UGA codon in the coding region of the messenger ribonucleic acid (mRNA). All selenoproteins contain Sec, the 21st amino acid, within their active sites [11,42,45]. In humans, 25 selenoproteins have been identified, and 24 of them exist as Sec-containing proteins in rodents. Selenoproteins exhibit a wide variety of tissue distribution and functions [41,46,47]. They can be found in the blood, liver, spleen, kidney, bowel, etc. [41]. They can play a crucial role in antioxidant action (e.g., glutathione peroxidases), the transport and storage of Se (SELENOP), redox signaling, thyroid hormone metabolism, protein folding, and others. Many members of the selenoprotein family function as enzymes involved in redox reactions [48,49], including glutathione peroxidases (GPXs), thioredoxin reductases (TRs), deiodinases (DIOs), and selenoprotein P (SEPP). Meanwhile, some are likely not enzymes themselves, and functions are gradually becoming better understood for these nonenzymatic members [12,44,46,50].

Se plays an important role in maintaining body health and can regulate body health through oxidative stress and immune regulation. Se usually exists in the form of selenite, Secys, SeMet, and selenate. Se is absorbed in the body mainly in the form of selenoproteins. GPX1 and GPX2 can regulate body health by mediating the production of reactive oxygen species. SELENOP can be expressed as a plasma transporter in various organs, and it is downregulated in cancer and IBD. DIO1 can affect the metabolism and activity of thyroid hormones. Loss of DIO1 contributes to renal carcinogenesis, and its induced expression protects cells against cancerous proliferation and migration. SELENOI regulates body health through the nervous system. Loss of SELENOI promotes neurodegenerative diseases such as Alzheimer’s disease and hereditary spastic paraplegia.

## 3. Se Is Involved in the Regulation of Intestine-Related Diseases

### 3.1. Se Is Associated with the Occurrence of Intestinal Cancer

Colorectal cancer (CRC) is a common malignancy that affects human health worldwide. It has been found that lower Se levels, usually due to lower Se intake, are associated with an increased risk of large intestine malignancies [37]. In contrast, an increase in Se intake was associated with a reduced risk of colorectal adenoma recurrence. Suitable Se levels contribute to personal wellness [1,11,46,51]. Organisms maintain their health through redox homeostasis. Reactive oxygen species and their metabolites are both important signal molecules, which can participate in the regulation of the signal transduction process, as well as the activation and inactivation of transcription factors and other target proteins. Selenoproteins contain several enzymes involved in cellular redox homeostasis, such as glutathione peroxidase and thioredoxin reductase family members [44,46]. It has often been shown that redox homeostasis in cancer cells is dysregulated, and the redox function of redox enzymes or selenoproteins with thioredoxin, such as folding, has a certain influence on the occurrence of colorectal cancer. Among glutathione peroxidases, GPX1 is the earliest known to be associated with a variety of human diseases, including cancer development and progression. GPX1 has been shown to be altered at both the protein and the mRNA levels in the tissues of CRC patients [51]. GPX1 mainly prevents cytotoxicity and inflammation to resist the tumor. Research indicated that GPX2, GPX4, and TXNRD3 were upregulated and that SELENOP, SELENOS, and GPX3, SELENOK were significantly downregulated in tumor tissues of CRC patients in Ireland compared to controls [50,52]. Knocking down SELENOH led to changes in the cell cycle, and abnormal cell proliferation was usually associated with dysregulation of the cell cycle. Additionally, SELENOH knockdown led to the growth of tumor xenotransplantation. Thus, the high expression of SELENOH in healthy proliferating cells as compared to more differentiated intestinal cells suggests a key role of SELENOH in controlling cell-cycle progression and intestinal homeostasis [53,54,55]. Genomic studies and animal models have indicated that Se intake influences the expression of selenoprotein genes and pathways key to colorectal carcinogenesis such as the antioxidant response, immune and inflammatory pathways (including NF-κB and Nrf2 signaling), the Wnt signaling pathway, protein synthesis, the regulation of eiF4E and p70S6 kinase, ribosomal proteins, and the mammalian target of rapamycin (mTOR) signaling pathway [56,57,58,59]. Importantly, deregulation of these signaling pathways has been associated with the carcinogenic process. A 15 kDa selenoprotein (Sep15) in colon cancer was evaluated by preparing and using mouse colon CT26 cells stably transfected with shRNA constructs targeting Sep15. The results of Northern and Western blot analyses showed that more than 90% of Sep15 was downregulated, proving that Sep15 has tissue specificity in influencing cellular proliferation [57,60]. SELENOP influenced inflammatory tumorigenesis by affecting genomic stability, the inflammatory microenvironment, and epithelial stem-cell functions [50,52]. Common selenoproteins associated with intestinal diseases are shown in Table 2.

### 3.2. Se Can Affect Inflammatory Bowel Diseases

Inflammatory bowel diseases (IBDs) are a group of chronic, nonspecific intestinal inflammatory diseases whose etiologies have not been clarified, including Ulcerative colitis (UC) and Crohn’s disease (CD) Ulcerative colitis is characterized by inflammation that is limited to the colon; it begins in the rectum, spreads proximally in a continuous fashion, and frequently involves the periappendiceal region. By contrast, Crohn’s disease involves any part of the gastrointestinal tract, most commonly the terminal ileum or the perianal region, in a noncontinuous fashion. Unlike UC, CD is commonly associated with complications such as strictures, abscesses, and fistulas [44,61]. Histologically, UC shows superficial inflammatory changes limited to the mucosa and submucosa with cryptitis and crypt abscesses. The microscopic features of CD include thickened submucosa, transmural inflammation, fissuring ulceration, and noncaseating granulomas [41,62]. Currently, there is no cure for IBD. Treatment of the disease is aimed at reducing debilitating symptoms to ensure long-term remission. For the treatment of IBD, anti-inflammatory steroids and immunosuppressants are commonly used [63]. In some extreme cases, a portion of the intestine can be removed as an alternative treatment. Although the etiology of IBD is unclear at present, recent studies have shown that an individual’s genetic susceptibility, external environment, intestinal microbiota, and immune response are all related to the occurrence of IBD [61,63,64].

Some epidemiological studies have indicated that Se levels are reduced in patients with these two types of IBD: UC and CD [6,7]. This is mainly manifested in the reduction in SELENOP (SEPP1) in the serum and the reduction in glutathione peroxidase activity in CD [13,14]. Similarly, SELENOS and SELENOK are also associated with inflammation and IBD [15,63]. Several experimental models of IBD and associated colon cancer have shown that Se and selenoproteins play a key role in microinflammation and tumor inflammation [56,57]. Studies have shown a correlation between intestinal NF-κB expression levels and IBD severity. Han et al. showed a correlation between NF-κB levels and histological score in colon samples before surgical resection of CD, with higher NF-κB levels leading to higher histological score [61]. Inhibiting NF-κB activation in DSS-induced colitis and proinflammatory cytokine secretion can prevent the onset of colitis [64]. NF-κB is also regulated by selenoproteins as a redox-sensitive transcription factor. Se supplementation after LPS stimulation of macrophages inhibits NF-κB phosphorylation and, thus, inhibits NF-κB activation [63,65]. Zhu et al. used Se nanoparticles coated with *Ulva lactuca* polysaccharide (ULP) to treat mice subjected to DSS-induced colitis, and they found that mice treated with Se nanoparticles showed a reduction in pathological characteristics, characterized by weight loss, lower disease activity index scores, and longer colon length, compared with untreated mice. The authors also found that, in DSS and the application of Se nanoparticles in mice, the activation of the NF-κB is restrained when UC and CD of the epithelial barrier are interfered with, and immune cells become more and more active, leading to epithelial cells, lymphocytes, and macrophages producing a reduction in inflammatory cytokines, such as IL-6 and TNFα [66]. Similarly, oxidative stress can be caused by damage to the gastrointestinal mucous membrane barrier, leading to the development of IBD; therefore, as antioxidants, selenoproteins could reduce IBD symptoms. During the period of IBD, oxidative stress can lead to NF-κB activation [58], such as GPX2 and SEPP1. In the case of IBD, where NF-κB is highly activated, Se supplementation has therapeutic effects that confer beneficial functions by reducing NF-κB activation and creating a homeostatic environment in the intestine [62].

Peroxisomal proliferator-activated receptor gamma, PPARγ, is a nuclear hormone receptor involved in biosynthesis and lipid metabolism. The expression level of PPAR in special pathways is affected by Se [48,61,66]. As with NF-κB, PPARγ is also involved in the regulation of colon inflammation. In fact, it is a key receptor that is heavily expressed in colonic epithelial cells; NF-κB expression was found to be increased during IBD, but the opposite was observed in the case of PPARγ. Interestingly, PPARγ was reduced more in patients with UC than in those with CD [67]. Several clinical and preclinical studies have reported the role and potential mechanisms of PPARγ in inflammatory diseases. Studies using human subjects revealed that colon biopsies from two patient groups showed lower PPARγ mRNA expression in both UC and CD samples and higher PPAR mRNA expression in patients with ulcerative colitis [46,48]. Interestingly, PPARγ expression or its deficiency is primarily confined to epithelial cells and is associated with activity in ulcerative colitis [51]. Experimental colitis can be relieved by increasing PPARγ expression through PPARγ agonists. Research has suggested that Se can increase both PPARγ and its ligand, which can prevent the IBD pathology involved in promoting the production of inflammatory cytokines [41,44,50].

## 4. Disease-Related Mechanisms of Se Regulation

### 4.1. Se Acts as an Antioxidant

Phosphatidylethanolamine is a glycerolipid that, together with phosphatidylcholine, makes up more than half of the total phospholipids in eukaryotic cell membranes.

SELENOI is the final step in the formation of phosphatidylethanolamine from CDP- ethanolamine in the Kennedy pathway. Mustafa et al. showed that decreasing the enzymatic activity of SELENOI impeded the last step of phosphatidylethanolamine synthesis [68]. SELENOI loss leads to abnormalities in the Kennedy pathway that are characteristic of motor neuron disease, including spasms, weakness, increased tendon reflexes, and upward plantar reflexes, a condition known as hereditary spastic paraplegia [69].

Selenoproteins have a strong antioxidant capacity, among which the glutathione peroxidase family has been proven to alleviate diseases by enhancing the antioxidant capacity of the body [1,40,69]. Studies have shown that GPXs can reduce the production of reactive oxygen species by increasing the intestinal antioxidant level, which can reduce LPS-induced colitis [41]. In the study of neurodegenerative diseases, Se has been used as an antioxidant to reduce oxidative stress in the CNS and ultimately reduce the effects of AD [39]. SELENOP can affect Aβ and phosphorylated Tau aggregation because transporters contribute to the synthesis of the antioxidant selenoprotein in the brain and signal transduction via neuron ApoER2 [52,69]. SELENOK (11 kDa) plays an important role in promoting the effective Ca^2+^ influx during the activation of immune cells. Enhancing the migration and phagocytosis of microglial cells is of importance to reduce the risk of neurodegenerative diseases, such as AD and Parkinson’s disease (PD) [70,71].

### 4.2. Se Has an Effect on the Diversity of Intestinal Microbiota

In recent years, more and more evidence has shown that intestinal redox status is closely related to intestinal flora. Previous studies have stated that the composition of the gut microbiota influences the Se content in mice, and that host Se status also modulates the composition of gut microbiota. Diets with different Se content could affect the microbial composition in rats [72]. Dietary Se increases the diversity of the microbiome, and changes occur in both Se deficiency and Se supplementation. Dietary Se can affect the composition and colonization of intestinal flora in the gastrointestinal tract, thus affecting host Se status and Se proteome expression. The disruption of the integrity of the gut flora can lead to diseases, such as IBD and cancer. Se supplementation, which increases the diversity of the gut microbiota, may be beneficial in preventing colon cancer [47,73,74]. Researchers have previously investigated the ability of nano-Se to improve the delivery of Se to birds and have characterized the resulting modifications of the intestinal microbiota [72]. The abundance of some beneficial bacteria, such as *Lactobacillus* sp. and *Faecalibacterium prausnitzii*, was increased. The quantity of butyric acid in different gut sections was increased. Butyric acid is a primary energy source for intestinal colonocytes and can promote good gut health [60]. Research revealed the role of gut microflora in the metabolism of Se in the host animal. Several inorganic and organic selenocompounds were metabolized to SeMet by intestinal microflora, and SeMet was incorporated into general bacterial proteins. The gut microflora accumulated SeMet-containing proteins that were available as an Se pool for the host animal [74,75].

### 4.3. Se Regulates the Nervous System

The gut–brain axis is a bidirectional neurohumoral communication system between the CNS and the enteric nervous system (ENS). Obviously, there is increasing evidence of a direct connection between gastrointestinal function and the brain. For instance, traumatic brain injury activated the gut–brain axis and increased intestinal permeability [76,77]. On the other hand, changes in gut microbial composition during neurodevelopment in early life may be detrimental for the CNS and lead to neurological disorders in later life [78]. Se deficiency is detrimental for the gut barrier function, inducing a disordered intestinal immune response in mice. Furthermore, it can decrease the levels of neuroactive substances, such as serotonin and melatonin, which are involved in the gut–brain axis [75,79]. Se is critical for the physiological functions of the brain with neuroprotective effects. Selenoproteins are found to play roles in the regulation of neurodegenerative disorders such as Huntington’s disease. Epidemiological studies have indicated that Se benefits neurological function and cognitive performance. Insufficient levels of Se in the brain and the disruption of Se homeostasis will cause damage to brain function and may expedite neuronal loss and dysfunction. Studies illustrated that selenoproteins alleviated dopaminergic neuron loss in multiple rodent models of acute toxicity [80,81,82]. Matthew summarized studies conducted in cell cultures and rodent models, showing that Se can reduce oxidative stress, prevent neurodegeneration, and counter cellular signaling mechanisms known to be dysregulated in certain disease states. In addition, Se deficiency and inadequate selenoprotein expression impair innate and adaptive immune responses, with an increase in inflammatory cytokines, especially at the colonic level [83]. At present, although Se can affect intestinal health through the immune system, few studies have linked Se with intestinal nerves. Perhaps future studies can focus on the interaction between Se and intestinal nerves, thus further revealing the mechanism regulating intestinal health.

SELENOP changes in the substantia nigra (SN) and the vagus nucleus in Parkinson’s disease showed that SELENOP plays a role in the dopaminergic transmission of SN neurons and the black striatum, and it may be important for the survival of these neurons in PD [12,46,50,52,84]. In a model of methamphetamine-induced Parkinson’s disease, Se-rich mice demonstrated a protective effect with reduced striatal dopamine consumption compared with Se-deficient mice. 6-Hydroxydopamine (6-OHDA) is a neurotoxin specific to catecholamine neurons in the central and peripheral nervous systems. By upregulating antioxidant status and decreasing dopamine loss in a dose-dependent manner, Se can help to mitigate the progression of neurodegeneration in Parkinson’s disease [50,52,79]. In one study, SELENOP knockout mice developed ataxia and a clumsy gait by the third week of life. Mice that were fed a Se-deficient diet lost motor coordination, which was prevented when fed with an Se-deficient diet containing at least 0.1 ppm of Se. This suggests that Se plays a crucial role in maintaining motor coordination in mice. SELENOP and other Se compounds are reported to interact with peroxynitrites and, thus, have a protective effect on motor neurons [82,83,84]. SELENOP is involved in protecting human astrocytes from oxidative damage by studying astrocytes and SELENOP [12]. Experimental autoimmune encephalomyelitis (EAE) is a T-cell-mediated inflammatory and demyelinating disease of the CNS. Diphenyldiselenide has strong antioxidant activity and neuroprotective effects [85]. In addition, this selenide can reduce characteristic histological changes, while it can also reduce the response of T cells to brain-pathogenic myelin basic protein in vivo and in vitro, thus playing a role in the treatment of EAE. Se protects against lipid peroxidation, which in turn protects phospholipid precursors and lipid-containing membrane components from peroxidation [82,85].

Se stimulates membrane synthesis by increasing the activity of choline phosphotransferase, a key enzyme in the Kennedy pathway that catalyzes the formation of PC from diacylglycerol and cytidine diphosphate (CDP) choline, which is an important component of biofilms and is essential for maintaining body health [85,86]. Phosphatidylethanolamine (PE) is the second most abundant phospholipid in the lipid bilayer of organelle membranes in mammals. It has many functions, such as membrane fusion. SELENOI is an enzyme involved in the synthesis of PE and plasmenyl-PE in two metabolic pathways [87,88]. SELENOI deficiency leads to a variety of pathologies, including reduced myelin sheath and neural development, as well as cellular damage that maintains normal homeostasis of ether-bound phospholipids [70]. SELENOI plays an essential role in myelination and neurodevelopment and in the maintenance of normal homeostasis of ether-bound phospholipids in the human body [89,90,91,92].

The intestinal microbiota is important in maintaining neurodevelopment and myelination, although the exact mechanisms are not yet understood. The microbiome and specific microbial metabolites (such as butyrate) act as key modulators of myelin gene expression in CNS [76]. Butyrate is produced by beneficial bacteria and is an important energy source for colon cells. Oral butyrate enhances myelination in the central nervous system of mice [86]. In the case of adult intestinal microbiota disorders, antibiotic administration in neonates results in oligodendrocyte damage and myelin changes in the prefrontal cortex, with behavioral deficits that can be prevented by SCFA supplementation [78,79].

### 4.4. Se Regulates Endothelial Function

**Figure 2:**
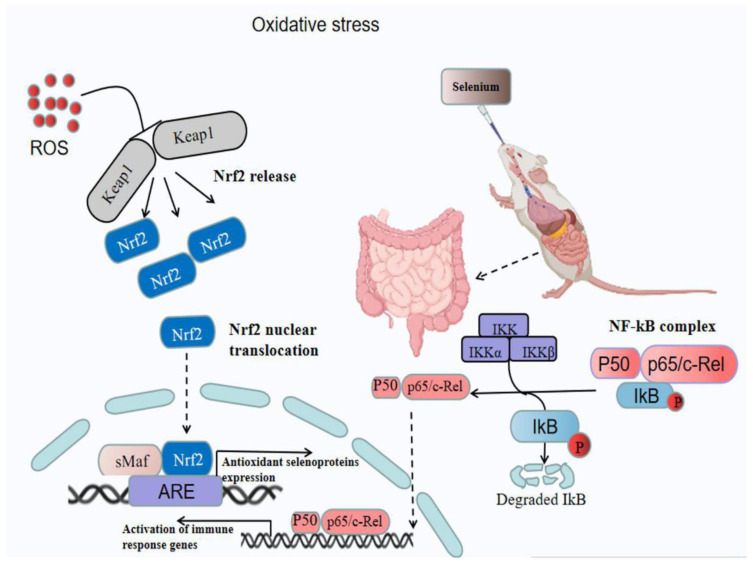
Selenium affects the typical pathways of intestinal disease.

The endothelium acts as a second barrier during inflammation, and there is a wide range of evidence showing that inflammatory conditions of the intestinal mucosa lead to a compromised epithelial barrier. Epithelial barrier integrity has been discussed as a pivotal function in protection against invading enteric pathogens. Disruption of an intact intestinal epithelial barrier has been found to play significant pathophysiologic roles in IBD [93]. The intestinal epithelium, which exhibits both organ-specific and immune functions, has been recognized as playing a crucial role in the pathogenesis of IBD. Epithelial cells form a cell barrier lining the GI tract between the host and various organisms, which is necessary for maintaining mucosal homeostasis. The barrier layer is maintained by tight junctions, adherens junctions, and desmosomes. Paneth cells, goblet cells, absorptive cells, and enteroendocrine cells are the four major secretory cell types in IEC populations. During mucosal inflammation, IECs can produce excess ROS; this ROS overload can injure cytoskeleton proteins and generate alterations in the tight junctions and epithelial permeability in IECs, ultimately contributing to barrier disruption [94,95]. Loss of both GPX1 and GPX2 can worsen colitis [11,48]. Furthermore, decreases in SELENOP contribute to inflammatory tumorigenesis by reducing redox capacity, enhancing proliferation of epithelial cells, and modulating immune cell polarization toward a pro-tumorigenic phenotype. Previous studies proved that colonic epithelial-derived SELENOP is the source of antioxidant-mediated protection in colitis-associated cancer [50,84,96]. The mechanism of selenium regulating intestinal diseases through classical pathways under oxidative stress can be seen in Figure 2.

The mechanism of Se’s involvement diseases is detailed in Figure 2.

Under oxidative stress, inflammation can cause intestinal damage through a variety of signaling pathways. The figure explains that oral Se supplementation can alleviate intestinal inflammation through the Nrf2 pathway and the NF-κB pathway. Under the condition of oxidative stress, Nrf2 and Keap are uncoupled and moved into the nucleus, resulting in the release of Nrf2. Oral Se can reduce the expression of Nrf2 and alleviate the damage caused by oxidative stress. NF-κB is a classic antioxidant pathway. Under normal conditions, NF-κB exists in the cytoplasm in a potential inactive state, and it combines with inhibitory factor IKB to form a trimer P50–P65–IKB. Under oxidative stress, NF-κB complexes dissociate and increase the level of NF-κB. Oral Se can inhibit the expression of NF-κB and alleviate the damage caused by oxidative stress.

## 5. Nano-Se Alleviates the Degree of Disease

### 5.1. Comparison of Modification of Nano-Se Particles by Different Biomacromolecules

Se nanoparticles (SeNPs) have anti-inflammatory activity and low toxicity, which improve when decorated with natural biological compounds. Due to the outstanding biological activity and biological safety of nano-level elemental Se particles, they have attracted more and more attention [97,98,99]. However, Se nanoparticles are usually unstable in the absence of biomacromolecules as stabilizers; thus, researchers have tried to prepare stable Se nanomaterials using proteins, polysaccharides, and other macromolecules as stabilizers or dispersants. Kaur et al. used bovine serum albumin (BSA) as a shape-guiding agent to synthesize shape-controlled BSA–Se bio-conjugated semiconductor nanomaterials to improve the function of nano-Se particles [100]. Song et al. used bacteria-modified nano-Se particles to assess the antioxidant capacity of specific pathways, and the results showed that BNS is a promising Se species with potential applications in the treatment of oxidative stress-related intestinal diseases [101]. Hyperbranched polysaccharides, as a kind of natural polysaccharide, not only participate in various life activities of cells, but also participate in the mineralization process of plants and animals. Using natural hyperbranched polysaccharide (HBP) as a stabilizer and capping agent, water-dispersing Se nanoparticles were prepared. HBP can not only play the role of capping to form nanoparticles, but can also provide a shell layer to prevent the agglomeration of nanoparticles [102]. Sulfated *Ganoderma lucidum* polysaccharides (SPS) can be used as a modifier and stabilizer to modify nano-Se particles to prevent the agglomeration of Se particles. Wang et al. modified Se nanoparticles with sulfated *Ganoderma* polysaccharides to investigate the effect of the SeNPs–SPS complex on immune regulation. The results showed that the SeNPs–SPS complex has significant anti-inflammatory activity, and this mechanism is partly due to inhibition of the activation of MAPK, NF-κB, JNK1/2, and p38 [103].

In brief, from the perspectives of environmental protection, energy saving, and the preparation method, polysaccharides have excellent biocompatibility, low cost, high biodegradability, and nontoxicity, with an active hydroxy and complex branching structure. They can modify the nanoparticle interface, control the growth of nanoparticles, and stabilize the nanoparticle solution. Therefore, polysaccharides are more suitable as stabilizers of Se nanoparticles than other materials, such as proteins and polyphenols [103,104,105,106].

### 5.2. Surface Modification of Nano-Se Particles Alleviates the Degree of Disease

A number of studies have found that the use of polysaccharides in the surface modification of chemically synthesized SeNPs with antioxidants or anticancer agents can significantly improve the antioxidant and anticancer functions of SeNPs [101,103,107]. Song et al. studied the effectiveness of the Nrf2 antioxidant pathway in alleviating the Diquat-induced damage to the intestinal barrier by using a new form of Se nanoparticle; furthermore, by comparing BNS with chemically synthesized nano-Se particles and SeMet, it was proven that BNS exhibited stronger antioxidant activity, which could protect against intestinal barrier injury [101]. In addition, Zhu et al. demonstrated that nanoparticles modified with *Ulva lactuca* polysaccharides reduced colitis in mice induced by DSS via the NF-κB pathway, and that nanoparticles modified with *Ulva lactuca* polysaccharides significantly increased colon GSH content and enhanced antioxidant capacity. Moreover, they could reduce the production of MDA to alleviate DSS-induced colitis in mice [66]. Zhang et al. demonstrated that selenomethylselenocysteine (SeMSC) increased the activity of GPX, TrxR, and GST with the same effect as selenomethylselenocysteine (SeMSC), but with much lower toxicity, suggesting that nano-Se can be used as a chemical. Potential chemoprophylaxis can significantly reduce the risk of Se poisoning [107]. Zhang et al. used ATP to modify the surface of nano-Se and demonstrated that this ATP-modified nano-Se particle can enhance cell permeability and selective apoptosis-induced activity, as well as induce the caspase-mediated apoptosis of HepG2 cells with the participation of ROS production through mitochondrial dysfunction. The results indicated that using ATP as a surface modifier of SeNPs is a novel strategy to achieve the anticancer synergistic effect [108]. Yang et al. used spirulina polysaccharides (SPS) to modify the surface of SeNPs to investigate the effects of cell uptake capacity and anticancer activity. The results showed that SPS-modified SeNPs enhanced the cell uptake and anticancer effect of nano-Se, providing a reference for the treatment of cancer [109]. In summary, nano-Se as a new preparation has low toxicity and high bioavailability, which may provide it with advantages in the prevention and treatment of related diseases.

### 5.3. Comparison of Nano-Se and Conventional Se Supplements

Se occurs in various oxidation states of +6, +4, 0, and −2. Se usually exists in the form of selenite, Secys, SeMet, and selenate. Generally, these forms can be used as feed additives. Se plays a vital role in multiple organs of the body, such as the brain, liver, kidney, and intestine, mainly in the form of selenoproteins [110,111]. There are certain differences in selenoproteins in different organs. Organic and inorganic forms of Se are not metabolized in the same way. Inorganic forms of Se experience reductive metabolism through a number of intermediate steps, leading to the generation of hydrogen selenide (H_2_Se), which serves as the precursor for the synthesis of essential selenoproteins. The metabolism of organic Se differs compared to that of inorganic varieties. Organic Se mainly acts in the form of SeMet [112]. SeMet is also metabolized intracellularly to the same key intermediate H_2_Se; however, SeMet can be directly incorporated into bacterial proteins and, thus, is protected from further metabolism. Both the organic and the inorganic forms of Se are useful in the body to produce selenoproteins. Inorganic Se combines with other food components during digestion to form insoluble complexes, reducing its absorption, while organic Se undergoes amino-acid uptake mechanisms in the intestine, increasing its transportation across the intestinal wall. There is enough evidence to indicate that the bioavailability of oral organic Se is greater than that of inorganic Se [110,112]. Nano-Se particles, such as zerovalent nano-Se and Se polysaccharides, are Se supplements that have a lower risk of toxicity and higher levels of bioavailability. In animals including fish, dietary nano-Se has been shown to improve the growth performance and antioxidant defense system, because it is a more easily available source of Se [111,113]. Nano-Se prepared via the chemical method is simple and the cost is low, but the disadvantage is that the particles tend to coagulate and have slight toxicity. The use of biological macromolecules such as proteins, polyphenols, polysaccharides, and other modifications can reduce toxicity, as well as improve antioxidant capacity and other biological functions [114,115].

## 6. Conclusions and Perspectives

Se is a trace element that is essential for human health. It acts as an antioxidant and immunomodulator, and it is involved in the control of specific endocrine pathways in both humans and animals. Se is also necessary in the livestock and poultry industry. In production applications, Se is often used as an additive in broiler feed in the poultry industry to improve immunity and the overall health of the animals, in order to boost production performance and economic value. As one of the important threats to health, IBD is regulated by many factors. The enteric nervous system, known as the second brain, can mediate IBD. In recent years, researchers have paid more and more attention to the importance of trace elements in the body. As a necessary trace element in the body, there have been studies showing that changes in Se level can affect IBD. It is not clear whether there is a specific association between Se levels and intestinal nerves as a factor affecting IBD, and future research may explain this association.

As a new type of Se preparation, SeNPs have low toxicity and good anti-inflammatory activity. When surface modification is applied with natural compounds, their anti-inflammatory and antioxidant functions are enhanced. At present, natural polysaccharides such as chitosan, spirulina polysaccharides (SPS), and other surface modifiers have been used to modify SeNPs to obtain better anti-inflammatory, antioxidative, and anticancer effects. Compared with inorganic Se and organic Se, modified nano-Se particles have higher bioavailability. However, the specific mechanism of nano-Se particles in the treatment of diseases is still unclear. In the future, through in-depth research on nano-Se, the treatment mechanism of some diseases such as enteritis, neurodegenerative diseases, and cancer will be elaborated in detail.

## Figures and Tables

**Table 1:** The distribution of Se content in environment and foods.

Source	Average Content	References
Soil	0.01–2 mg/kg	[22,25,27,28]
Water	0.02–742 μg/L	[20,25,28]
Cereal	0.1–10 μg/g	[21,23,27]
Vegetables	0.0008–5.37 mg/kg	[24,25,28]
Fruits	<0.01 μg/g	[20,21,23,27]
Animal-based foods	0.3–0.5 μg/g	[18,24,27,28]

**Table 2:** Function of selenoproteins associated with intestinal diseases.

Selenoproteins	Abbreviation	Functions	Change	Distribution	References
Glutathione peroxidase 1	GPX1	Antioxidant enzymes	↓	Cytosol, mitochondria; widely expressed	[2,9,46]
Glutathione peroxidase2	GPX2	Antioxidant protection	↑	Cytosol, ER; gastrointestinal tissue, liver	[2,11,49,51]
Glutathione peroxidase3	CPX3	Antioxidant enzymes	↓	Secreted, plasma, gastrointestinal tissue	[12,46,51]
Thioredoxin reductase 1	TXNRD1	Reduce thioredoxin	↑	Cytosol, nucleus; widely distributed	[46,48,49]
Selenoprotein H	SELENOH	Nucleolar oxidoreductase	↑	Nucleus	[53,54,55]
Selenoprotein P	SELENOP	transport and store Se	↓	Secreted, cytosol; plasma, widely expressed, brain, liver, testes	[50,52]
15 kDa selenoprotein	Sep15	Regulates cell proliferation and cell cycle	↑	Brain, prostate, testis, liver, kidney	[57,60]

Selenoprotein expression levels are altered in colon cancer. ↑ represents an increase in the level of selenoprotein. Similarly, ↓ represents a decrease in the level of selenoprotein.

## Data Availability

Not applicable.

## Abbreviations

IBD, inflammation bowel disease; ROS, reactive oxygen species; AA, arachidonic acid; ACh, acetylcholine; AD, Alzheimer’s disease; BNS, biogenic nano-selenium; BSA, bovine serum albumin; CD, Crohn’s disease; CDP, cytidine diphosphate; CLA, conjugated linoleic acid; CNS, central nervous system; CRC, colorectal cancer; DHA, docosahexaenoic acid; DIOs, deiodinases; DSS, dextran sulfate sodium; ENS, enteric nervous system; EGCs, enteric glial cells; EAE, experimental autoimmune encephalomyelitis; EPA, eicosapentaenoic acid; GPXs, glutathione peroxidases; HBP, hyperbranched polysaccharide; HPGDS, hematopoietic PGD2 synthase; IBD, inflammatory bowel disease; IEB, intestinal epithelial barrier; KD, Keshan disease; KBD, Kashin–Beck disease; MDA, malondialdehyde; mRNA, messenger ribonucleic acid; NF-κB, nuclear factor kappa B; PC, phosphatidyl choline; PD, Parkinson’s disease; PE, phosphatidylethanolamine; PGD2, prostaglandin D2; PPARγ, peroxisomal proliferator-activated receptor γ; SCFA, short-chain fatty acid; Sec, selenocysteine; SeCys, selenocysteine; SEPP, SELENOP, selenoprotein P; SELENOH, selenoprotein H; SELENOK, selenoprotein K; SELENOS, selenoprotein S; SeNPs, selenium nanoparticles; SeMet, selenomethionine; SeMSC, selenomethylselenocysteine; SPS, spirulina polysaccharide; TNFα, tumor necrosis factor α; TRs, thioredoxin reductases; UC, ulcerative colitis; ULP, *Ulva lactuca* polysaccharide; VIP, vasoactive intestinal peptide.

## References

[1] Prabhu K.S., Lei X.G. (2016). Selenium. Adv. Nutr..

[2] Rayman M.P. (2012). Selenium and human health. Lancet.

[3] Schwarz K., Foltz C.M. (1999). Selenium as an integral part of factor 3 against dietary necrotic liver degeneration. Nutrition.

[4] Zhou H., Wang T., Li D. (2018). Prevention of Keshan Disease by Selenium Supplementation: A Systematic Review and Meta-analysis. Biol. Trace Elem. Res..

[5] Hou J., Zhu L., Sum D. (2021). Association of selenium levels with the prevention and control of Keshan disease: A cross-sectional study. J. Trace Elem. Med. Biol..

[6] Avery J.C., Hoffmann P.R. (2018). Selenium, Selenoproteins, and Immunity. Nutrients.

[7] Rayman M.P. (2000). The importance of selenium to human health. Lancet.

[8] Foster H.D., Zhang L. (1995). Longevity and selenium deficiency: Evidence from the People’s Republic of China. Sci. Total Environ..

[9] Roman M., Jitaru P., Barbante C. (2014). Selenium biochemistry and its role for human health. Metallomics.

[10] Rayman M.P. (2008). Food-chain selenium and human health: Emphasis on intake. Br. J. Nutr..

[11] Rayman M.P. (2020). Selenium intake, status, and health: A complex relationship. Hormones.

[12] Papp L.V., Lu J., Holmgren A. (2007). From selenium to selenoproteins: Synthesis, identity, and their role in human health. Antioxid. Redox Signal..

[13] Zhang Y.Z., Li Y.Y. (2014). Inflammatory bowel disease: Pathogenesis. World J. Gastroenterol..

[14] Khor B., Gardet A., Xavier R.J. (2011). Genetics and pathogenesis of inflammatory bowel disease. Nature.

[15] Hodson R. (2016). Inflammatory bowel disease. Nature.

[16] Labunskyy V.M., Hatfield D.L., Gladyshev V.N. (2014). Selenoproteins: Molecular pathways and physiological roles. Physiol. Rev..

[17] Reeves M.A., Hoffmann P.R. (2009). The human selenoproteome: Recent insights into functions and regulation. Cell Mol. Life Sci..

[18] Dinh Q.T., Cui Z., Huang J. (2018). Selenium distribution in the Chinese environment and its relationship with human health: A review. Environ. Int..

[19] Kim J.H., Dong Y.K. (2020). Comparison of toxic effects of dietary organic or inorganic selenium and prediction of selenium intake and tissue selenium concentrations in broiler chickens using feather selenium concentrations. Poult. Sci..

[20] Emmanuelle B., Virginie M., Fabienne S. (2012). Selenium exposure in subjects living in areas with high selenium concentrated drinking water: Results of a French integrated exposure assessment survey. Environ. Int..

[21] Weekley C.M., Harris H.H. (2013). Which form is that? The importance of selenium speciation and metabolism in the prevention and treatment of disease. Chem. Soc. Rev..

[22] Hadrup N., Ravn-Haren G. (2021). Absorption, distribution, metabolism and excretion (ADME) of oral selenium from organic and inorganic sources: A review. J. Trace Elem. Med. Biol..

[23] Panchal S.K., Wanyonyi S., Brown L. (2017). Selenium, Vanadium, and Chromium as Micronutrients to Improve Metabolic Syndrome. Curr. Hypertens. Rep..

[24] Mehdi Y., Hornick J.L., Istasse L., Dufrasne I. (2013). Selenium in the environment, metabolism and involvement in body functions. Molecules.

[25] Tan J., Zhu W., Wang W. (2002). Selenium in soil and endemic diseases in China. Sci. Total Environ..

[26] Hesketh J. (2008). Nutrigenomics and selenium: Gene expression patterns, physiological targets, and genetics. Annu. Rev. Nutr..

[27] Shahid M., Niazi N.K., Rashid M.I. (2018). A critical review of selenium biogeochemical behavior in soil-plant system with an inference to human health. Environ. Pollut..

[28] Ullah H., Liu G., Ahmad R. (2019). A comprehensive review on environmental transformation of selenium: Recent advances and research perspectives. Environ. Geochem. Health.

[29] Hatfield D.L., Tsuji P.A., Carlson B.A. (2014). Selenium and selenocysteine: Roles in cancer, health, and development. Trends Biochem. Sci..

[30] Yao Y., Pei F., Kang P. (2011). Selenium, iodine, and the relation with Kashin-Beck disease. Nutrition.

[31] Driscoll D.M., Copeland P.R. (2003). Mechanism and regulation of selenoprotein synthesis. Annu. Rev. Nutr..

[32] Vindry C., Ohlmann T., Chavatte L. (2018). Translation regulation of mammalian selenoproteins. Biochim. Biophys. Acta Gen. Subj..

[33] Reszka E., Jablonska E., Gromadzinska J. (2012). Relevance of selenoprotein transcripts for selenium status in humans. Genes Nutr..

[34] Short S.P., Pilat J.M., Williams C.S. (2018). Roles for selenium and selenoprotein P in the development, progression, and prevention of intestinal disease. Free Radic. Biol. Med..

[35] Cardoso B.R., Roberts B.R., Bush A.I. (2015). Selenium, selenoproteins and neurodegenerative diseases. Metallomics.

[36] Pillai R., Uyehara-Lock J.H., Bellinger F.P. (2014). Selenium and selenoprotein function in brain disorders. IUBMB Life.

[37] Hughes D.J., Kunická T., Schomburg L. (2018). Expression of Selenoprotein Genes and Association with Selenium Status in Colorectal Adenoma and Colorectal Cancer. Nutrients.

[38] Shchedrina V.A., Zhang Y., Labunskyy V.M. (2010). Structure–Function Relations, Physiological Roles, and Evolution of Mammalian ER-Resident Selenoproteins. Antioxid. Redox Signal..

[39] Steinbrenner H., Speckmann B., Klotz L.O. (2016). Selenoproteins: Antioxidant selenoenzymes and beyond. Arch. Biochem. Biophys..

[40] Murawaki Y., Tsuchiya H., Kanbe T. (2008). Aberrant expression of selenoproteins in the progression of colorectal cancer. Cancer Lett..

[41] Ying H., Zhang Y. (2019). Systems Biology of Selenium and Complex Disease. Biol. Trace Elem. Res..

[42] Ha H.Y., Alfulaij N., Seale L.A. (2019). From Selenium Absorption to Selenoprotein Degradation. Biol. Trace Elem. Res..

[43] Ramos G.P., Papadakis K.A. (2019). Mechanisms of Disease: Inflammatory Bowel Diseases. Mayo Clin. Proc..

[44] Geerling B.J., Badart-Smook A., Stockbrügger R.W. (2000). Comprehensive nutritional status in recently diagnosed patients with inflammatory bowel disease compared with population controls. Eur. J. Clin. Nutr..

[45] Nettleford S.K., Prabhu K.S. (2018). Selenium and Selenoproteins in Gut Inflammation-A Review. Antioxidants.

[46] Kudva A.K., Shay A.E., Prabhu K.S. (2015). Selenium and inflammatory bowel disease. Am. J. Physiol. Gastrointest. Liver Physiol..

[47] Santesmasses D., Mariotti M., Gladyshev V.N. (2020). Bioinformatics of Selenoproteins. Antioxid. Redox Signal..

[48] Castro A.T., Navarro-Alarcón M., Quesada G.J. (2016). Ulcerative Colitis and Crohn’s Disease Are Associated with Decreased Serum Selenium Concentrations and Increased Cardiovascular Risk. Nutrients.

[49] Jäger S., Stange E.F., Wehkamp J. (2013). Inflammatory bowel disease: An impaired barrier disease. Langenbeck’s Arch. Surg..

[50] Kipp A.P. (2020). Selenium in colorectal and differentiated thyroid cancer. Hormones.

[51] Peters K.M., Carlson B.A., Gladyshev V.N. (2018). Selenoproteins in colon cancer. Free Radic. Biol. Med..

[52] Barrett C.W., Reddy V.K., Short S.P. (2015). Selenoprotein P influences colitis-induced tumorigenesis by mediating stemness and oxidative damage. J. Clin. Investig..

[53] Ma Y.M., Guo Y.Z., Ibeanu G. (2017). Overexpression of selenoprotein H prevents mitochondrial dynamic imbalance induced by glutamate exposure. Int. J. Biol. Sci..

[54] Qazi I.H., Angel C., Yang H. (2018). Selenium, Selenoproteins, and Female Reproduction: A Review. Molecules.

[55] Bertz M., Kühn K., Koeberle S.C. (2018). Selenoprotein H controls cell cycle progression and proliferation of human colorectal cancer cells. Free Radic. Biol. Med..

[56] Fedirko V., Jenab M., Hughes D.J. (2019). Association of Selenoprotein and Selenium Pathway Genotypes with Risk of Colorectal Cancer and Interaction with Selenium Status. Nutrients.

[57] Méplan C., Hesketh J. (2012). The influence of selenium and selenoprotein gene variants on colorectal cancer risk. Mutagenesis.

[58] Barrett C.W., Short S.P., Williams C.S. (2017). Selenoproteins and oxidative stress-induced inflammatory tumorigenesis in the gut. Cell Mol. Life Sci..

[59] Steinbrenner H., Speckmann B., Sies H. (2013). Toward understanding success and failures in the use of selenium for cancer prevention. Antioxid. Redox Signal..

[60] Irons R., Tsuji P.A., Davis C.D. (2010). Deficiency in the 15-kDa selenoprotein inhibits tumorigenicity and metastasis of colon cancer cells. Cancer Prev. Res..

[61] Han Y.M., Koh J., Kim J.W. (2017). NF-kappa B activation correlates with disease phenotype in Crohn’s disease. PLoS ONE.

[62] Barrett C.W., Singh K., Motley A.K. (2013). Dietary selenium deficiency exacerbates DSS-induced epithelial injury and AOM/DSS-induced tumorigenesis. PLoS ONE.

[63] Huang L.J., Mao X.T., Li Y.Y. (2021). Multiomics analyses reveal a critical role of selenium in controlling T cell differentiation in Crohn’s disease. Immunity.

[64] Atreya I., Atreya R., Neurath M.F. (2008). NF-kappaB in inflammatory bowel disease. J. Intern. Med..

[65] Koeberle S.C., Gollowitzer A., Laoukili J. (2020). Distinct and overlapping functions of glutathione peroxidases 1 and 2 in limiting NF-κB-driven inflammation through redox-active mechanisms. Redox Biol..

[66] Zhu C., Zhang S., Song C. (2017). Selenium nanoparticles decorated with Ulva lactuca polysaccharide potentially attenuate colitis by inhibiting NF-κB mediated hyper inflammation. J. Nanobiotechnol..

[67] Song Y., Kurose A., Li R. (2021). Ablation of Selenbp1 Alters Lipid Metabolism via the Pparα Pathway in Mouse Kidney. Int. J. Mol. Sci..

[68] Arias-Borrego A., Callejón-Leblic B., Calatayud M., Gómez-Ariza J.L., Collado M.C., García-Barrera T. (2019). Insights into cancer and neurodegenerative diseases through selenoproteins and the connection with gut microbiota-current analytical methodologies. Expert Rev. Proteom..

[69] Bellinger F.P., Raman A.V., Rueli R.H. (2012). Changes in selenoprotein P in substantia nigra and putamen in Parkinson’s disease. J. Parkinsons Dis..

[70] Fredericks G.J., Hoffmann P.R. (2015). Selenoprotein K and protein palmitoylation. Antioxid. Redox Signal..

[71] Lee J.H., Jang J.K., Ko K.Y. (2019). Degradation of selenoprotein S and selenoprotein K through PPARγ-mediated ubiquitination is required for adipocyte differentiation. Cell Death Differ..

[72] Foong J.P.P., Hung L.Y., Poon S. (2020). Early life interaction between the microbiota and the enteric nervous system. Am. J. Physiol. Gastrointest. Liver Physiol..

[73] Takahashi K., Suzuki N., Ogra Y. (2020). Effect of gut microflora on nutritional availability of selenium. Food Chem..

[74] Lv C.H., Wang T., Liao S.F. (2015). Effects of dietary supplementation of selenium-enriched probiotics on production performance and intestinal microbiota of weanling piglets raised under high ambient temperature. J. Anim. Physiol. Anim. Nutr..

[75] Li Z., Dong Y., Xu S. (2021). Organic Selenium Increased Gilts Antioxidant Capacity, Immune Function, and Changed Intestinal Microbiota. Front Microbiol..

[76] Keogh C.E., Kim D.H.J., Pusceddu M.M. (2021). Myelin as a regulator of development of the microbiota-gut-brain axis. Brain Behav. Immun..

[77] Li X.J., You X.Y., Wang C.Y. (2020). Bidirectional Brain-gut-microbiota Axis in increased intestinal permeability induced by central nervous system injury. CNS Neurosci. Ther..

[78] Stakenborg N., Boeckxstaens G.E. (2021). Bioelectronics in the brain-gut axis: Focus on inflammatory bowel disease (IBD). Int. Immunol..

[79] Chen J., Berry M.J. (2003). Selenium and selenoproteins in the brain and brain diseases. J. Neurochem..

[80] Wang X., Wu J., Liu X. (2021). Engineered liposomes targeting the gut–CNS Axis for comprehensive therapy of spinal cord injury. J. Control Release.

[81] Zhang Z.H., Song G.L. (2021). Roles of Selenoproteins in Brain Function and the Potential Mechanism of Selenium in Alzheimer’s Disease. Front Neurosci..

[82] Solovyev N., Blume B. (2021). Selenium at the Neural Barriers: A Review. Front. Neurosci..

[83] Pitts M.W., Byrns C.N., Berry M.J. (2014). Selenoproteins in nervous system development and function. Biol. Trace Elem. Res..

[84] Steinbrenner H., Alili L., Bilgic E. (2006). Involvement of selenoprotein P in protection of human astrocytes from oxidative damage. Free Radic. Biol Med..

[85] Ahmed M.Y., Al-Khayat A., Al-Murshedi F. (2017). A mutation of EPT1 (SELENOI) underlies a new disorder of Kennedy pathway phospholipid biosynthesis. Brain.

[86] Horibata Y., Elpeleg O., Eran A. (2018). EPT1 (selenoprotein I) is critical for the neural development and maintenance of plasmalogen in humans. J. Lipid Res..

[87] Kieliszek M. (2019). Selenium–Fascinating Microelement, Properties and Sources in Food. Molecules.

[88] Avery J.C., Yamazaki Y., Hoffmann F.W. (2020). Selenoprotein I is essential for murine embryogenesis. Arch. Biochem. Biophys..

[89] Bourassa M.W., Alim I., Bultman S.J. (2016). Butyrate, neuroepigenetics and the gut microbiome: Can a high fiber diet improve brain health?. Neurosci. Lett..

[90] Chanaday N.L., Bem A.F., Roth G.A. (2011). Effect of diphenyl diselenide on the development of experimental autoimmune encephalomyelitis. Neurochem. Int..

[91] Ma C., Hoffmann F.W., Marciel M.P. (2021). Upregulated ethanolamine phospholipid synthesis via selenoprotein I is required for effective metabolic reprogramming during T cell activation. Mol. Metab..

[92] Fairweather-Tait S.J., Bao Y., Broadley M.R. (2011). Selenium in human health and disease. Antioxid. Redox Signal..

[93] Tian T., Wang Z., Zhang J. (2017). Pathomechanisms of Oxidative Stress in Inflammatory Bowel Disease and Potential Antioxidant Therapies. Oxid. Med. Cell Longev..

[94] Fukata M., Arditi M. (2013). The role of pattern recognition receptors in intestinal inflammation. Mucosal Immunol..

[95] Garrett W.S., Gordon J.I., Glimcher L.H. (2010). Homeostasis and inflammation in the intestine. Cell.

[96] Huang J.Q., Ren F.Z., Jiang Y.Y. (2015). Selenoproteins protect against avian nutritional muscular dystrophy by metabolizing peroxides and regulating redox/apoptotic signaling. Free Radic. Biol. Med..

[97] El-Sayed E.R., Abdelhakim H.K., Ahmed A.S. (2020). Solid-state fermentation for enhanced production of selenium nanoparticles by gamma-irradiated Monascus purpureus and their biological evaluation and photocatalytic activities. Bioprocess. Biosyst. Eng..

[98] Hu S., Hu W., Li Y. (2020). Construction and structure-activity mechanism of polysaccharide nano-selenium carrier. Carbohydr. Polym..

[99] Hosnedlova B., Kepinska M., Skalickova S. (2018). Nano-selenium and its nanomedicine applications: A critical review. Int. J. Nanomed..

[100] Kaur G., Iqbal M., Bakshi M.S. (2009). Biomineralization of Fine Selenium Crystalline Rods and Amorphous Spheres. J. Phys. Chem. C.

[101] Song D., Cheng Y., Li X. (2017). Biogenic Nanoselenium Particles Effectively Attenuate Oxidative Stress-Induced Intestinal Epithelial Barrier Injury by Activating the Nrf2 Antioxidant Pathway. ACS Appl. Mater. Interfaces.

[102] Zhang Y., Wang J., Zhang L. (2010). Creation of Highly Stable Selenium Nanoparticles Capped with Hyperbranched Polysaccharide in Water. Langmuir.

[103] Wang J., Zhang Y., Yuan Y. (2014). Immunomodulatory of selenium nano-particles decorated by sulfated Ganoderma lucidum polysaccharides. Food Chem. Toxicol..

[104] Yu S., Wang Y., Zhang W. (2016). pH-Assisted surface functionalization of selenium nanoparticles with curcumin to achieve enhanced cancer chemopreventive activity. RSC Adv..

[105] Rao L., Ma Y., Zhuang M. (2014). Chitosan-Decorated Selenium Nanoparticles as Protein Carriers to Improve the In Vivo Half-Life of the Peptide Therapeutic BAY 55-9837 for Type 2 Diabetes Mellitus. Int. J. Nanomed..

[106] Zhang X., Yan H., Ma L. (2020). Preparation and characterization of selenium nanoparticles decorated by *Spirulina platensis* polysaccharide. J. Food Biochem..

[107] Zhang J., Wang X., Xu T. (2008). Elemental selenium at nano size (Nano-Se) as a potential chemopreventive agent with reduced risk of selenium toxicity: Comparison with se-methylselenocysteine in mice. Toxicol. Sci..

[108] Zhang Y., Li X., Zhi H. (2013). Enhancement of cell permeabilization apoptosis-inducing activity of selenium nanoparticles by ATP surface decoration. Nanomedicine.

[109] Fang Y., Tang Q., Zhong X. (2012). Surface decoration by Spirulina polysaccharide enhances the cellular uptake and anticancer efficacy of selenium nanoparticles. Int. J. Nanomed..

[110] McKelvey S.M., Horgan K.A., Murphy R.A. (2015). Chemical form of selenium differentially influences DNA repair pathways following exposure to lead nitrate. J. Trace Elem. Med. Biol..

[111] Kumar A., Prasad K.S. (2021). Role of nano-selenium in health and environment. J. Biotechnol..

[112] Lynch S.J., Horgan K.A., Walls D. (2017). Selenium Source Impacts Protection of Porcine Jejunal Epithelial Cells from Cadmium-Induced DNA Damage, with Maximum Protection Exhibited with Yeast-Derived Selenium Compounds. Biol. Trace Elem. Res..

[113] Gangadoo S., Dinev I., Stanley D. (2020). Nanoparticles of selenium as high bioavailable and non-toxic supplement alternatives for broiler chickens. Environ. Sci. Pollut. Res..

[114] Surai P.F., Kochish I.I. (2020). Food for thought: Nano-selenium in poultry nutrition and health. Anim. Health Res. Rev..

[115] Kuperman R.G., Checkai R.T., Simini M. (2010). Selenium toxicity to survival and reproduction of Collembola and Enchytraeids in a sandy loam soil. Environ. Toxicol. Chem..

